# DOVE: An Infectious Disease Outbreak Statistics Visualization System

**DOI:** 10.1109/ACCESS.2018.2867030

**Published:** 2018-08-24

**Authors:** Miran Lee, Jong Wook Kim, Beakcheol Jang

**Affiliations:** Department of Computer ScienceSangmyung UniversitySeoul03016South Korea

**Keywords:** Infectious disease information system, infectious disease outbreak statistics, infectious disease, Korea Centers for Disease Control & Prevention

## Abstract

Humans are susceptible to various infectious diseases. However, humanity still has limited responses to emergent and recurrent infectious diseases. Recent developments in medical technology have led to various vaccines being developed, but these vaccines typically require a considerable amount of time to counter infectious diseases. Therefore, one of the best methods to prevent infectious diseases is to continuously update our knowledge with useful information from infectious disease information systems and taking active steps to safeguard ourselves against infectious diseases. Some existing infectious disease information systems simply present infectious disease information in the form of text or transmit it via e-mail. Other systems provide data in the form of files or maps. Most existing systems display text-centric information regarding infectious disease outbreaks. Therefore, understanding infectious disease outbreak information at a glance is difficult for users. In this paper, we propose the infectious disease outbreak statistics visualization system, called to DOVE, which collects infectious disease outbreak statistics from the Korea Centers for Disease Control & Prevention and provides statistical charts with district, time, infectious disease, gender, and age data. Users can easily identify infectious disease outbreak statistics at a glance by simply entering the district, time, and name of an infectious disease into our system. Additionally, each statistical chart allows users to recognize the characteristics of an infectious disease and predict outbreaks by investigating the outbreak trends of that disease. We believe that our system provides effective information to help prevent infectious disease outbreaks. Our system is currently available on the web at http://www.epidemic.co.kr/statistics.

## Graphical abstract

User Interface of infectious Disease Outbreak Statistics Visualization System (DOVE).

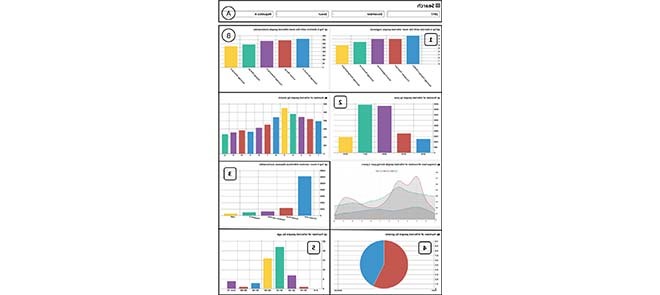

## I. Introduction

During the sixth century in Rome, approximately five million people died from smallpox. In the 14th century, China lost half its population to the plague, which then spread to Europe, resulting in the deaths of approximately 75 million people [1]–[3]. According to the World Health Organization (WHO), in 2003, 3,098 people were infected with severe acute respiratory syndrome (SARS) worldwide and 774 died [4], [5]. The number of deaths from the Hong Kong flu outbreak was 600 in 2017, with the total number of deaths being approximately twice the number of SARS deaths. Humans have always suffered from a variety of infectious diseases such as avian flu, swine flu, SARS, Ebola, and Zika. We still suffer not only from emergent infectious diseases but also recurring infectious diseases. Recent advances in medical technology have halted the spread of many infectious diseases through the development of appropriate vaccines. However, vaccine development requires a considerable amount of time.

One of the best ways to prevent the spread of infectious diseases is to continuously provide useful information regarding such diseases. Consequently, interest in infectious disease information systems has recently increased. An infectious disease information system is a system that continuously collects data related to the occurrences of infectious diseases, converts data into usable information through systematic analysis, and distributes the information to various individuals and agencies [6], [7].

### A. Contributions of This Paper

Based on the importance of disease control, many researchers have carried out wide-ranging studies on infectious disease information systems [8]–[33]. Many existing systems collect and analyze infectious disease information from news or media sources and then present it on the web in text format [13], [20], [23], [24], [27], [30]. Some systems send infectious disease information via e-mail or text messages [11]–[18]. Other systems also provide data in extensible markup language (XML), Excel spreadsheet (XLS), and comma-separated values (CSV) files and simply present the infectious disease information and corresponding risk levels utilizing a map or chart [19]–[32]. In other words, existing infectious disease information systems are text-centric. They do not include or only partially include visualized data. Therefore, users cannot easily understand information regarding infectious disease outbreaks at a glance.

In this paper, we propose a novel infectious disease outbreak information system, called to DOVE, which provides a variety of valuable information visualizations for infectious disease outbreaks. We collect infectious disease outbreak data from the Korea Centers for Disease Control & Prevention (KCDC). Currently, the KCDC only provides text data on infectious disease outbreaks, which is insufficient for effective transmission of infectious disease information. Utilizing these data, DOVE provides various effective charts containing infectious disease outbreak statistics sorted by district, time, infectious disease, gender, and age according to user input. The main benefits of DOVE are as follows:

• Users can see current infectious disease statistics at a glance through DOVE.
• Users can identify the characteristics of infectious diseases.
• Users can predict outbreaks of infectious diseases by investigating disease trends because DOVE provides not only current, but also past infectious disease outbreak statistics.
• DOVE is currently available on the web at http://www.epidemic.co.kr/statistics. It provides valuable information regarding infectious disease outbreaks to end users and helps researchers to study the characteristics of infectious disease outbreaks academically.

The remainder of this paper is organized as follows. In Section II, we discuss existing infectious disease information systems. In Section III, we describe the development environment and system architecture of DOVE. In Section IV, we present the results of implementing our system based on various statistical criteria (district, time, infectious disease, gender, and age). We also provide various examples demonstrating how users can get helpful information from DOVE. Finally, in Section V, we discuss final conclusions regarding the proposed system.

## II. Related Work

Disease information systems have been established in many parts of the world to provide useful disease information. In this section, we describe how different systems present disease information to users in detail.

The systems from [11]–[13] provide disease information via e-mails or text messages. In 1994, the United States began providing infectious disease outbreak information for the first time through the Program for Monitoring Emerging Diseases mail (ProMED-mail) [14], [15]. ProMED-mail sends infectious disease outbreak information via e-mail and is now available on the web. ProMED-mail procures information regarding infectious disease outbreaks by analyzing e-mails from its subscribers. It then filters this information through a review process, sends it to ProMED-mail subscribers via e-mail, and posts it on a website. On the website for ProMED-mail, users can retrieve infectious disease information by searching for keywords associated with an infectious disease, dates, or locations. ProMED-mail analyzes infectious disease information and presents the risk levels based on three colors of red, yellow, and green. Red indicates high risk, yellow indicates moderate risk, and green indicates low risk. The Global Public Health Intelligence Network (GPHIN) is a disease information system developed in Canada in collaboration with the WHO in 1997 [16], [17]. This system displays real-time data regarding various public-health-related information and diseases. The GPHIN collects articles related to diseases from the web and analyzes them. The GPHIN then delivers filtered information to users via e-mail. When the e-mails are delivered, the GPHIN provides customized information according to pre-collected user preferences. Additionally, the system provides web search functionality, allowing users to view article information collected by the GPHIN. The Medical Information System (MedIsys) collects infectious disease information from media sources and analyzes it. Users can receive the analyzed information via e-mail or SMS by searching for keywords or selecting countries. Finally, GETWELL is a system that provides disease information utilizing the search engine of a medical website [18]. GETWELL stores queries from the search engine in its database with search dates, analyzes the queries, and sends the resulting information to users via e-mail.

Some systems provide disease information in a variety of file types rather than via e-mail or text. Examples of such systems are the KCDC system [19], EpiSPIDER [20], Google Flu Trends [21], and Influenzanet [22]. The KCDC constructed an infectious disease portal system that provides infectious disease incident information sorted by disease, region, gender, and age in three file formats: XML, XLS, and CSV. EpiSPIDER is a disease information system that collects information in various forms, such as ProMED-mail, really simple syndication feeds, and social network services. EpiSPIDER provides users with disease outbreak information in the form of a CSV file. Google’s Flu Trends is a system that utilizes Google search data to presents information regarding influenza outbreaks. Users can download the information provided by Flu Trends in the form of a CSV file. Finally, Influenzanet is a system on the web that analyzes the prevalence of influenza-like illnesses. This system provides data on weekly outbreaks of influenza-like illnesses in a CSV file.

There are also disease information systems that utilize methods for providing information regarding disease outbreaks on a map. Typical examples are HealthMap [23], BioCaster [24], Google Flu Trends [25], [26], FluNet [27], and EpiSPIDER. HealthMap is a mutual disease information map based on the Google Maps API. HealthMap provides information regarding the occurrence of infectious diseases, primarily through geographic locations. This system collects infectious disease outbreak data in real time via Google, the news media, WHO, and infectious-disease-related websites. The filtered information includes the date of infectious disease outbreak, location where the infectious disease appeared, and other infectious disease information. The location of an outbreak is shown on a map. HealthMap calculates weights according to the date of occurrence, number of disease outbreaks, and location information, and it displays risk levels on a map. The aforementioned ProMED-mail also provides location information via the HealthMap in web-based systems. BioCaster also presents new keywords and information related to diseases filtered by month or week. Google Flu Trends analyzes influenza-related terms entered by users with regard to specific regions and then classifies influenza activity into five grades: intense, high, moderate, low, and minimal. The system then visualizes the grades on a map. Flunet is a web-based system implemented by the WHO to create a global influenza network. Flunet fetches weekly data from influenza centers in several countries, analyzes outbreak information, and fetches infectious disease information from national agencies and the media in real time. This system visualizes the risk of influenza in a country on a map. Finally, EpiSPIDER extracts subjects, dates, and location information related to diseases from data collected by news agencies and other sources. This system provides disease information for a year on a map.

Some systems also chart disease outbreak information [28]–[30]. Fluview in the United States charts influenza outbreaks as a weekly chart of patient or death statistics, or charts outbreaks by age groups utilizing these data. The aforementioned HealthMap system presents infectious disease trends for one-year periods in the country where an infectious disease occurred when a user searches for the infectious disease. BioCaster in Japan visualizes the statistics of infectious disease outbreaks on a chart sorted by country. MedISys in Europe provides information in three classes, namely high, medium, and low, based on the order of the most frequent infectious diseases in each country. The statistics in this system are based on the number of articles related to infectious diseases. GETWELL in Sweden saves and analyzes queries from a search engine with dates and aggregates queries that are analyzed weekly. The aggregated results are listed on a web page as a graph. The system presents the graph as a time series graph for specific queries. Epidemic.co.kr in South Korea presents DiTex, which extracts important disease related topics from news media and SNS sources, presents weekly trends in the form of graphs, and visualizes topics utilizing World Cloud [31]. It also collects disease related data from news media SNS sources and analyzes characteristics of news and SNS data [32]. Flunet distinguishes influenza viruses as Virus A (H3N2), Virus A (H1N1), Virus B, etc. [33]. It also presents the numbers of outbreaks for each virus as a weekly bar graph. Finally, Europe’s Influenzanet presents the outbreaks of each influenza-like illness as a line graph for each country [34]. One graph compares data from Influenzanet to data from the European Center for Disease Prevention and Control. This system allows users to predict outbreaks and compare outbreak rates across countries in Europe.

Most existing systems do not provide statistical charts or provide them in an incomplete form. In this paper, we propose a system that visualizes various types of infectious disease outbreak statistics based on user inputs.

## III. System Architecture

In this section, we describe the development environment and architecture of our system. Table 1 details the development environment for our system. Our system retrieves infectious disease statistics collected by the KCDC and stores them in our database. Our system visualizes the stored numerical data according to user requests. We implemented our system in the Eclipse Java EE Oxygen integrated development environment [35]. Eclipse is an open-source integrated development environment that supports various computer languages, including JAVA. We utilize JAVA [36], [37], HTML5 [38], CSS [39], and JQuery [40] in our system. First, we implemented our statistical program utilizing JAVA and created a layout that users can view on a web browser utilizing HTML5. We also applied styling to present charts on a web page utilizing CSS. We implemented asynchronous JavaScript and XML (AJAX) [41] data transfer via JQuery. On the server side, we utilize jetty-9.0.6 for our web server and Spring Framework 4 [42] for our application server. Jetty is a JAVA web server and JAVA servlet container. Spring Framework is an open-source application framework for supporting JAVA. This framework provides a variety of functions for dynamic implementation of websites. Our system utilizes the Spring Framework to interact with the database and store or retrieve data. The framework then responds to requests from the web server in an appropriate form. Finally, we utilize PostgreSQL 9.6 [43], [44] for our database. PostgreSQL is an object-relational database management system. It supports SQL [45] standard and complex queries better than MYSQL [46] and has superior performance.

**TABLE 1:** Development Environment

Category	Description
Data	KCDC
Integrated Development Environment(IDE)	Eclipse Jee Oxygen
Languages	JAVA, HTML5, CSS, JQuery
Web Server	jetty-9.0.6
Application Server	Spring Framework 4
DataBase	PostgreSQL 9.6

**FIGURE 1.**
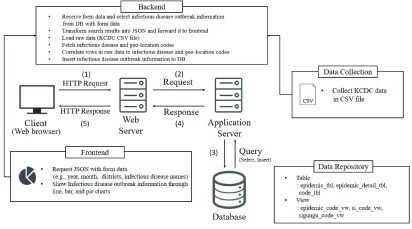
System architecture.

Figure 1 presents the architecture of our system implemented in the development environment described above. Our system consists of a client, web server, application server, and database. The client accesses figures for infectious disease statistics utilizing the web browser through the following processes: (1) The client sends a request to the web server including parameters specifying the information the user wishes to retrieve. Currently, the request method is HTTP GET [47]. The web server provides a web page implemented in HTML when a user receives a service request. (2) After confirming if the data selected by the user are appropriate based on the request transmitted from the client, the web server transfers the request to the application server. The application server is responsible for executing the program or generating a query through the database connection and receiving data. (3) The application server in our system generates a query for the received request and sends it to the database. The application server then receives the query results from the database. (4) The database sends the query results to the web server. (5) Finally, the web server adds the data to the body of the HTTP response and responds to the client.

Our system performs functions such as data collection, data repository, frontend operations, and backend operations. First, for data collection, we collect data regarding infectious diseases by district, gender, and age from the KCDC and store it in CSV file format. CSV files are comma-separated text data [48]. The data repository stores infectious disease statistics in two main tables with three views. Table 2 lists the detailed descriptions of the tables required to implement our system.

**TABLE 2:** Detailed Description of the Tables

Table	Detail
epidemic_tbl	Number of infected persons per each district and each infectious disease
epidemic_detail_tbl	Number of infected persons by age and gender of each infectious disease by district
code_tbl	Groupcode, Unique code and name of Si, Sigungu, infectious disease, upper group code
epidemic_code_vw	Infectious disease code, name, URL information
si_code_vw	Si-code, unit of Si (geo-location codes)
sigungu_code_vw	Sigungu-code, unit of Sigungu (geo-location codes)

**FIGURE 2.**
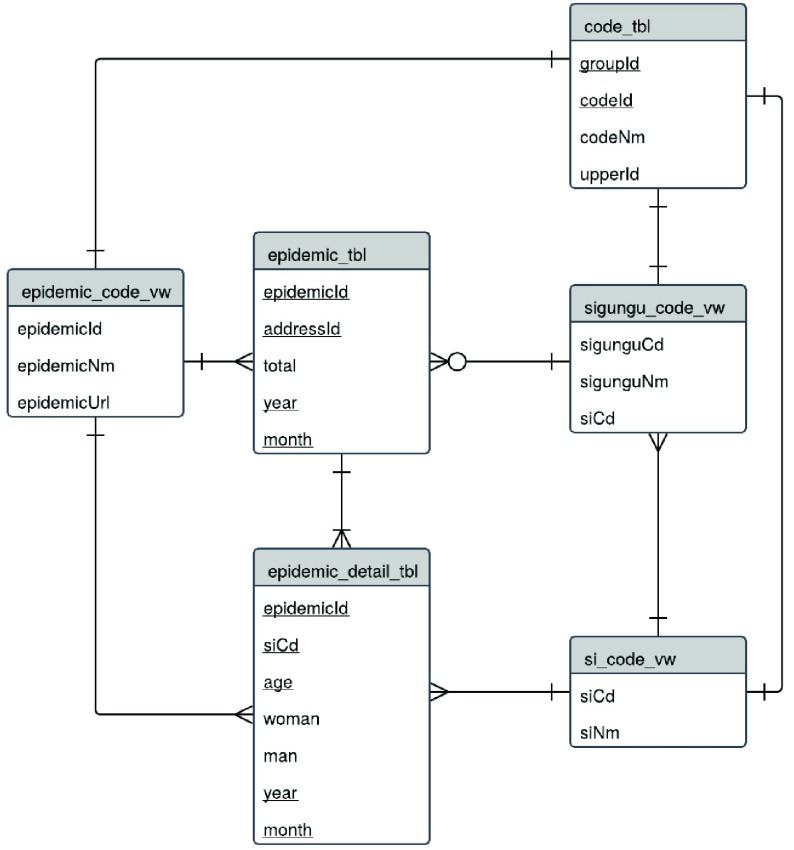
Entity relationship diagram.

Figure 2 presents the entity relationship (ER) diagram that defines the relationships between each table in our system. The ER diagram is a visual representation of tables in a database utilizing an object-relational model [49].

The backend converts the results of a user request into JavaScript Object Notation (JSON) [50], [51]. JSON is a light-weight data transfer type that consists of a collection of name/value pairs. The backend first receives a client request. It then retrieves the search form data selected by the client and verifies that the data type is appropriate. Next, it creates a Select query, sends it to the database, gets the results of the input from the client, and sends the results back to the client. The backend not only sends the result of a request to a client, but also processes infectious disease statistics collected by the KCDC and stores them in our database. Our system utilizes the Apache Commons

Library [52] to load data from CSV files and imports the infectious disease and geo-location code line-by-line to map the names of infectious diseases to the names of districts. When mapping is complete, the backend creates an Insert query, transfers the query to the database, and saves the numerical data.

The frontend provides an interface with a search form and chart area to display selected results. The backend transforms keywords (e.g., year, month, district, infectious disease name) from the search form selected by the user into JSON form and transmits them to the web server. We utilize AJAX to transfer data to the web server. AJAX is a web development technique for building asynchronous web applications and frontend functions to visualize search results from a web server for users. We utilize Chart.js [53] for visualization. Chart.js is an open-source library that provides various functions for drawing charts. We draw charts by district, time, infectious disease, gender, and age in the form of bar, line, and pie charts.

## IV. Results

**FIGURE 3.**
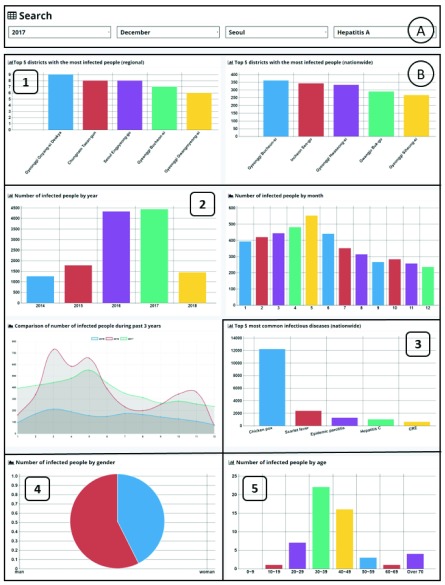
User Interface.

Our system presents the results of visualization of infectious disease outbreak queries based on five statistics criteria: district, time, infectious disease, gender, and age. We also describe how users can retrieve useful information through our system. Our system is currently available on the web through http://www.epidemic.co.kr/statistics. Figure 3 presents the web page that our system provides as a user interface. This web page includes the search form and eight charts. Part A in Figure 3 is the search form. Users can select the year, month, district, and infectious disease name. Part B is the block that displays infectious disease statistics charts according to user selections. Part B presents charts sorted by five statistical criteria. B-1 presents statistics by district, B-2 presents statistics by time, B-3 presents statistics by infectious disease, B-4 presents statistics by gender, and B-5 presents statistics by age. The details for each of the statistics are described below.

### A. District

The district is one of the most important factors affecting the outbreak of infectious diseases. Depending on the location of the district, the terrain and climate are different. In other words, the risk of infection for each infectious disease varies according to the characteristics of the district. In the Middle East, which has a desert-like climate, people encounter infectious diseases such as the Middle East respiratory syndrome coronavirus (MERS) and cholera. The outbreak of malaria in Korea was severe in Incheon, Gyeonggi, and Gangwon, which is close to North Korea. In Jeonnam, Gyeongnam, and Chungnam, the risk of scrub typhus is high.

**FIGURE 4.**
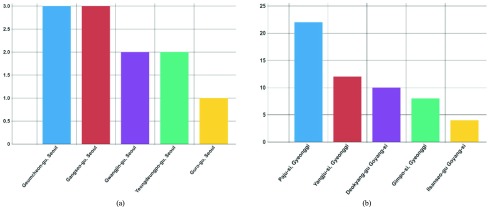
Top five districts with the most malaria-infected people (Regional). (a) Seoul. (b) Gyeonggi.

**FIGURE 5.**
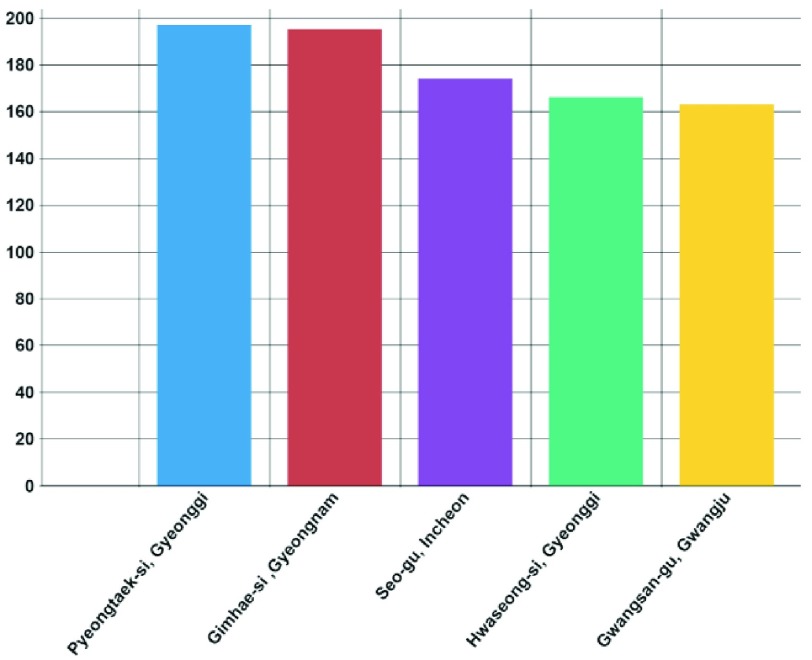
Top five districts with the most malaria-infected people (Nationwide).

Figure 4-a presents the top five districts in Seoul in July, 2017 in terms of risk for malaria infection. Figure 4-b presents the top five districts in Gyeonggi in terms of risk for malaria infection. Figure 5 presents the top five districts nationwide in July, 2017 in terms of risk for malaria infection. The x-axis represents the district, and the y-axis represents the infected population. This chart shows the user the district where the outbreak of the infectious disease was the most severe when they select the year, month, district, and infectious disease. Additionally, users can infer risky districts by comparing the charts from each district utilizing the infectious disease criteria. Figure 4 shows users the districts that are at high risk of infection in Seoul and Gyeonggi and helps by comparing the two charts to identify sensitive districts in terms of malaria occurrence. Figure 4-a presents the number of infections in each district with a high risk of infection representing three or fewer infections, whereas Figure 4-b- reveals that there were more than 20 infected people in a single district. The user can compare these two charts and predict that malaria will occur much more frequently in Gyeonggi than in Seoul.

### B. Time

Infection risks often change over time. In particular, countries located in mid-latitude temperate climatic zones are markedly seasonal. Korea, which has a seasonal climate, has a severe outbreak of infectious diseases such as vibrio vulnificus sepsis, malaria enterohemorrhagic, and escherichia coli in the summer, leptospirosis, rickettsia typhi, Japanese encephalitis, and scrub typhus in autumn, and hemorrhagic fever with renal syndrome in the winter.

**FIGURE 6.**
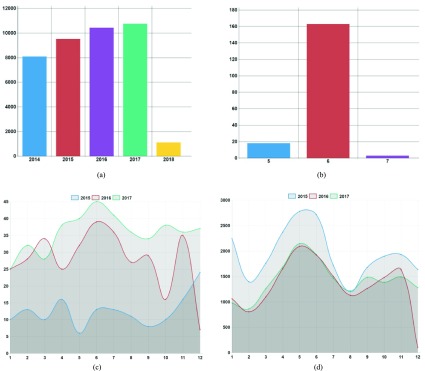
Number of infected people by time. (a) Number of infected people with Scrub typhus by year. (b) Number of infected people with MERS by month. (c) Comparison of the number of people infected with hepatitis B (2015–2017). (d) Comparison of the number of people infected with epidemic parotitis (2015–2017).

Figure 6-a is a graph showing yearly infection progress. It reveals the yearly infection progress of the scrub typhus disease at a glance. The x-axis represents the year (time) and the y-axis represents the number of infected people. It reveals that the number of scrub typhus infections has increased every year.

Based on time-specific statistical charts, one can not only easily identify infection trends, but also identify emergent infectious diseases. MERS became prevalent in Korea after the first confirmed patients in May, 2015 visited Saudi Arabia in 2012. Over the next three months, a total of 186 infections occurred and 37 people died, a mortality rate of nearly 20%.

Figure 6-b is a graph showing monthly outbreaks of MERS. The x-axis represents the month (time) and the y-axis represents the number of infected people. Figure 6-b reveals that MERS began to outbreak in May, 2015 and disappeared in August, 2015.

Infectious diseases affected by time are predictable because they exhibit similar patterns every year. Figure 6-c is a graph showing the outbreaks of hepatitis B over the past three years.

The x-axis represents the month (time) and the y-axis represents the number of infected people. Streptococcus pneumonia is an infectious disease that is not affected by time. It can be seen from

Figure 6-c that its trends are not consistent every year. Figure 6-d shows the outbreaks of epidemic parotitis over the past three years. Unlike Figure 6-c, Figure 6-d shows a similar pattern of infection every year. The trend charts in our system allow users to predict future infection trends.

### C. Infectious Disease

**FIGURE 7.**
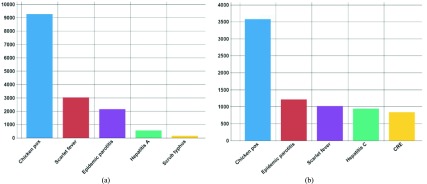
Top five most common infectious diseases (Nationwide) (a) May. (b) August.

The risk of infection varies according to the nature of the infectious disease. Figure 7 presents an example of the top five infectious diseases that occurred most frequently by year, month, and district. The x-axis is the infectious disease and the y-axis is the number of infected people. Figure 7-a is a chart showing the most common infectious diseases in May 2017. Figure 7-b shows the most common infectious diseases in August 2017. From Figure 7-a, the number of chickenpox infections during the month of May is approximately 9,000. From Figure 7-b, approximately 3,500 people were infected in the month of August. Figure 7 reveals that chickenpox is a common infectious disease in spring.

### D. Gender

Infectious diseases can also have different risks of infection depending on gender. Males are particularly at risk for acute hepatitis B, brucellosis, and vibrio vulnificus sepsis. The number of infected people with scrub typhus, rubella, and shigellosis was higher for women. Vibrio vulnificus sepsis is an infectious disease with a male-to-female ratio of 7:1, which represents a very large gender disparity.

**FIGURE 8.**
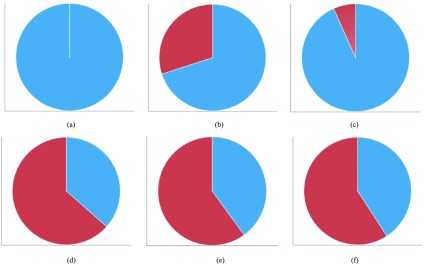
Number of infected people by gender (2015–2017). (a) Vibrio vulnificus sepsis, 2015. (b) Vibrio vulnificus sepsis, 2016. (c) Vibrio vulnificus sepsis, 2017. (d) Scrub typhus, 2015. (e) Scrub typhus, 2016. (f) Scrub typhus, 2017.

Figure 8 presents a chart showing gender statistics for vibrio vulnificus sepsis in August, (a) 2015, (b) 2016, and (c) 2017, and the same statistics for scrub typhus in October, (d) 2015, (e) 2016, and (f) 2017. Figures 8-a, 8-b, and 8-c reveal that vibrio vulnificus sepsis is more prevalent in males than females, whereas Figures 8-d, 8-e, and 8-f reveal that scrub typhus is more prevalent in females than males.

### E. Age

There are also age-related differences in the outbreak of infectious diseases. For example, the infection risks of pertussis, chickenpox, and enterohemorrhagic escherichia coli in young children, scarlet fever in children from 6–12 years of age, epidemic parotitis in children under 20 years of age, hepatitis A in adults from 20–40 years of age, scrub typhus and rickettsia typhi in adults over 50 years of age, and CJD/vCJD in elderly people are high. Chickenpox is one of the infectious diseases that occurs every year. In the case of chicken pox, approximately 90% of all infections occur in pediatric age groups.

**FIGURE 9.**
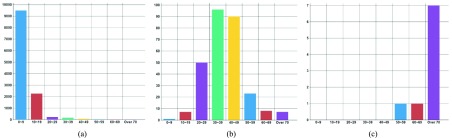
Number of infected people by age. (a) Chickenpox. (b) Hepatitis A. (c) CJD/vCJD.

Figure 9-a presents the age-specific statistics of chicken pox in December, 2017. The x-axis represents age and the y-axis represents the number of infected people. The proportion of infected people aged 0–9 years is remarkably high. Figure 9-b presents the age-specific statistics of hepatitis A in October, 2017. One can see that hepatitis A is more common in the 20–40 year age group than in children or the elderly. Figure 9-c presents the age-specific statistics of CJD/vCJD in October, 2017. This chart reveals that CJD/vCJD is an overwhelmingly common infectious disease in the elderly.

## V. Conclusion

Humans are susceptible to both emergent infectious diseases and recurring existing infectious diseases. With recent advances in medical technology, humans have overcome many diseases by developing vaccines. However, vaccine development requires a considerable amount of time. Therefore, until vaccines are developed, a temporary method to help to prevent infectious diseases is necessary. Most existing infectious disease information systems display only text-centric information with regard to infectious disease outbreaks. Therefore, grasping infectious disease outbreak information at a glance is difficult for users. The infectious disease information system proposed in this paper provides infection outbreak information through various forms of charts. We described our system configuration and showcased various implementation results in this paper. Our system, DOVE visualizes statistical data by district, time, infectious disease, gender, and age through a web interface. Users can not only infer the characteristics of infectious diseases from various charts but also predict their occurrence based on district, time, gender, and age. We believe that DOVE can aid people in easily identifying and preventing infectious diseases. DOVE is currently live on the web at http://www.epidemic.co.kr/statistics.
